# Remdesivir (GS-5734) Impedes Enterovirus Replication Through Viral RNA Synthesis Inhibition

**DOI:** 10.3389/fmicb.2020.01105

**Published:** 2020-06-12

**Authors:** Wei Ye, Min Yao, Yangchao Dong, Chuantao Ye, Dan Wang, He Liu, Hongwei Ma, Hui Zhang, Libin Qi, Yuewu Yang, Yuan Wang, Liang Zhang, Linfeng Cheng, Xin Lv, Zhikai Xu, Yingfeng Lei, Fanglin Zhang

**Affiliations:** ^1^Department of Microbiology, School of Preclinical Medicine, Fourth Military Medical University, Xi’an, China; ^2^Department of Infectious Diseases, Tangdu Hospital, Fourth Military Medical University, Xi’an, China; ^3^Second Affiliated Hospital, Xi’an Medical University, Xi’an, China; ^4^Cadet Brigade, School of Preclinical Medicine, Fourth Military Medical University, Xi’an, China

**Keywords:** Remdesivir (GS-5734), antivirals, EV71, vRNA, cRNA, enterovirus

## Abstract

Human enteroviruses are responsible for diverse diseases, from mild respiratory symptoms to fatal neurological complications. Currently, no registered antivirals have been approved for clinical therapy. Thus, a therapeutic agent for the enterovirus-related disease is urgently needed. Remdesivir (GS-5734) is a novel monophosphoramidate adenosine analog prodrug that exhibits potent antiviral activity against diverse RNA virus families, including positive-sense *Coronaviridae* and *Flaviviridae* and negative-sense *Filoviridae*, *Paramyxoviridae*, and *Pneumoviridae*. Currently, remdesivir is under phase 3 clinical development for disease COVID-19 treatment. Here, we found that remdesivir impeded both EV71 viral RNA (vRNA) and complementary (cRNA) synthesis, indicating that EV71 replication is inhibited by the triphosphate (TP) form of remdesivir. Moreover, remdesivir showed potent antiviral activity against diverse enteroviruses. These data extend the remdesivir antiviral activity to enteroviruses and indicate that remdesivir is a promising antiviral treatment for EV71 and other enterovirus infections.

## Introduction

Human enteroviruses, family *Picornaviridae*, genus *enterovirus*, are non-enveloped, single-stranded RNA viruses. Enteroviruses include a large number of viruses and are characterized into four species (A∼D) responsible for diverse diseases, including poliovirus (PV), which causes poliomyelitis; some coxsackieviruses (CVs), and echovirus, which cause myocarditis; and enterovirus 70 (EV70), which causes enteroviral conjunctivitis. The most prevalent enteroviruses, causing hand, foot and mouth disease (HFMD), are endemic worldwide, and the top two pathogens are coxsackie A16 virus (CAV16), and enterovirus 71 (EV71). EV71 was first identified in 1969 in California, United States (Schmidt et al., 1974) and is now a reemerging pathogen worldwide responsible for HFMD and other fatal neurological diseases, including meningitis, neurogenic pulmonary edema, acute paralysis, and reduced cognitive function. Since a massive outbreak in 2008, HFMD has become widespread in China, with over 2,000,000 cases annually. The pediatric population is most affected, mainly preschool children, and is at the highest risk of developing severe EV71-associated complications.

The approval of an inactivated EV71 vaccine in China has provided a specific prevention method for HFMD. However, the vaccination coverage rate is relatively low. Moreover, a lack of effective therapy or treatment still poses a severe threat to children’s health and social stability in China. Hence, finding safe and effective therapeutics against EV71 and other enteroviruses is an urgent need.

Nucleoside analogs, which target viral RNA-dependent RNA polymerases (RdRps) to inhibit viral transcription and replication, have recently achieved great success. Targeting non-structural protein 5 (NS5) of hepatitis C virus (HCV) makes this “ghost” disease curable. One type of nucleoside analog, a 1’-cyano-substituted adenine C-nucleoside ribose analog (Nuc), has been found to inhibit the replication of multiple viruses, including HCV, dengue virus (DENV), coronavirus (CoV), Nipah virus (NiV), and Ebola virus (EBOV) (Cho et al., 2012). Remdesivir (GS-5734) is a phosphoramidate prodrug of Nuc that inhibits EBOV and multiple CoVs with submicromolar half maximal effective concentration (EC_50_) values. Also, remdesivir inhibits numerous members within *Paramyxoviridae*, such as respiratory syncytial virus (RSV), measles virus (MV), mumps virus (MuV), human parainfluenza virus type 3 (hPIV3), human metapneumovirus (hMPV), Hendra virus (HeV), and NiV, with potent efficacy (Lo et al., 2017; Brown et al., 2019). Currently, the SARS-CoV-2, formally known as the 2019 novel coronavirus (2019-nCoV) is expanding throughout the globe, and remdesivir has been found to be effective in inhibiting SARS-CoV-2 replication *in vitro* (Wang et al., 2020) and relieved the symptoms of the first American case of SARS-CoV-2 infection (Holshue et al., 2020). Now, phase 3 clinical trials for remdesivir have been initiated to combat the SARS-CoV-2 pandemic. The antiviral mechanism of remdesivir is mediated through delayed chain termination of nascent viral RNA by targeting the viral RdRp. Considering the conservation of the key motif within RdRps, it is reasonable to speculate that remdesivir exhibits similar efficacy for enterovirus infection treatment. Here, we found that remdesivir could inhibit EV71 replication after virus entry and inhibit viral complementary RNA (cRNA) synthesis. Additionally, remdesivir inhibited coxsackievirus B3 (CVB3) and EV70 at promising rates. Overall, our results expand the antiviral spectrum of remdesivir and provide new evidence for the development of this antiviral as an effective anti-enterovirus therapeutic.

## Materials and Methods

### Cells, Viruses, Antibodies, and Regents

HeLa [American Type Culture Collection (ATCC), Manassas, VA, United States; CCL-2], was cultured in Dulbecco’s modified Eagle’s medium (DMEM; Sigma-Aldrich, St. Louis, MO, United States) supplemented with 10% fetal bovine serum (FBS; Sigma-Aldrich) with 5% CO_2_ at 37°C.

EV71 (strain 87-2008 Xi’an Shaanxi, GenBank accession no. HM003207.1) was obtained from the Xi’an Centre for Disease Control and Prevention (Xi’an, Shaanxi, China) as previously indicated (Deng et al., 2012). As the virus propagated in the laboratory for ten years, the genome was sequenced and submitted to NCBI under accession number MK904809.

Rabbit Enterovirus 71 VP2 antibody was purchased from GeneTex (GTX132340, Hsinchu, China). Mouse monoclonal double-stranded RNA (dsRNA) antibody J2 was obtained from SCICONS (10010500, Szirák, Hungary). Anti-GAPDH mouse monoclonal antibody (mAb) was purchased from Sangon (D190090, Shanghai, China).

Remdesivir (GS-5734, HY-104077, and purity 99.74%) was purchased from MedChemExpress (NJ, United States).

### Cytotoxicity Assay

Cytotoxicity assays were conducted following an established protocol (Ye et al., 2019). Briefly, HeLa cells were seeded in 96-well plates at 1.0 × 10^4^ cells per well and incubated at 37°C with 5% CO_2_ overnight. The original culture medium was discarded, and the cells were washed twice with Dulbecco’s phosphate-buffered saline (DPBS). Then, medium with vehicle control or antivirals at different concentrations was added to the wells, and samples were cultured for an additional 24 h. After the medium was removed, 100 μl of DMEM and 10 μl of Cell Counting Kit-8 (CCK8) solution (Yeasen, Shanghai, China) were added to the wells, and the cells were cultured for 4 h in the dark. The plate was shaken for 1 min, and the absorbance (A) was measured at 450 nm using a BioTek Synergy HT microplate reader. Cell viability was calculated using the following formula: Cell viability = [(As-Ab)/(Ac-Ab)] × 100%, where As denotes the absorbance of the experimental wells containing cells, medium, CCK8 solution, and drug; Ac denotes the absorbance of the control wells containing the same components as the As wells but without the drug; and Ab indicates the absorbance of the blank wells, containing only medium and CCK8 solution.

### Quantitative Reverse Transcription PCR (qRT-PCR) and EV71 cRNA Detection

HeLa cells were infected with EV71 (MOI = 1) for 1 h, and the inoculum was removed after virus adsorption. Then, the culture medium with remdesivir at different concentrations was added. After 24 h of treatment, cellular RNA was isolated and subjected to reverse transcription and subsequent qRT-PCR. Total cellular RNA was extracted using TRNzol Universal (#DP424, TIANGEN), and RNA concentration was determined with an Epoch microplate spectrophotometer (BioTek, Winooski, VT, United States). Reverse transcription (RT) was then performed with a Hifair^®^ III 1st Strand cDNA Synthesis Kit (Yeasen, Shanghai, China) according to instructions provided by the manufacturer. Briefly, equal amounts of total RNA (e.g., 5 μg) from cells treated with different concentrations of remdesivir were added to 3 μl 5 × gDNA Digester Mix and supplemented with RNase-free ddH_2_O to a final volume of 15 μl. The reaction mixtures were incubated at 42°C for 2 min. After incubation, 2 μl 10 × Hifair^®^ III Super Buffer, 1 μl Hifair^®^ III RT Enzyme Mix, and 1 μl oligo (dT)18 (50 μM) were added to a final volume of 20 μl with RNase-free ddH_2_O. Then, the mixture was reacted at 25°C for 5 min, followed by 55°C for 15 min, and 85°C for 5 min. The final reaction mixture was subjected to qRT-PCR performed using Hieff^®^ qPCR SYBR^®^ Green Master Mix (Yeasen) on a CFX96 Real-Time system (Bio-Rad, Hercules, CA, United States). Briefly, 10 μl of Hieff^®^ qPCR SYBR Green Master Mix, 0.4 μl forward primer (10 μM), and 0.4 μl reverse primer (10 μM) targeting corresponding genes were mixed with RNase-free ddH_2_O to a final volume of 20 μl. The qRT-PCR reaction was performed as a two-step method: after denaturation for 5 min, 40 cycles of 95°C for 10 s for denaturation and 60°C for 30 s for annealing/extension reaction were performed. A melting curve was constructed following the default setting of the CFX96 Real-Time system. The mRNA expression level of each target gene was normalized to the corresponding β-actin expression level. The primers used for gene amplification are listed in Table 1.

**TABLE 1:** Primers used for qRT-PCR.

Primer	Sequence (5′- 3′)
qhActin-F1379	AGCGAGCATCCCCCAAAGTT
qhActin-R1663	GGGCACGAAGGCTCATCATT
qEV71-F	GCAGCCCAAAAGAACTTCAC
qEV71-R	ATTTCAGCAGCTTGGAGTGC
qEV71-cRNA-F	GGTAAGCAAGAATATGAGGA
qEV71-cRNA-R	GCTAGCTTCAGCTAGGCATC
qEV71-cRNA-RT-R	GCTAGCTTCAGCTAGGCATCTTT
	TTTTTTGCTATTCTGGTTATAA
qCVB3-F	GATTTTGTGCTTTGTGTCAGC
qCVB3-R	GTATCTGCTGGTACAACCTGTG
qEV70-F	GAGGGATTCACCAGACATTG
qEV70-R	CTCTGCAGTACCATGCATA

### Immunofluorescence Assay (IFA)

Immunofluorescence assays (IFA) were conducted as previously described (Ye et al., 2015). Cells were seeded in 24-well plates at a confluence of 60–70%. After adherence, the cells were infected with EV71 at an MOI of 1 for 1 h, with rocking every 15 min. After absorption, the inoculum was removed, and medium with remdesivir at varying concentrations was added. At 24 h post infection (hpi), the cells were subjected to an IFA following an established protocol. Briefly, cells were fixed with 4% paraformaldehyde for 15 min, followed by permeabilization with 0.5% Triton X-100. Cells were incubated with primary antibody (J2) at 4°C overnight and with fluorescein-conjugated secondary antibody at 37°C for 2 h. Hoechst 33258 was used to stain cell nuclei, and the samples were imaged with an BX60 fluorescence microscope (Olympus, Tokyo, Japan).

### Western Blot Analysis

Cells were seeded in 6-well plates at a confluence of 60–70%. Then, the cells were infected with EV71, and the drug was added as described for the IFA. At 24 hpi, the cells were washed twice with DPBS and lysed with RIPA buffer (Beyotime, P0013C, or P0013D). Samples were quantified (BCA kit, Thermo Fisher Scientific, Waltham, MA, United States), and 40 μg aliquots of each cell lysate were boiled for 10 min. The lysates were subjected to 10% SDS-PAGE and transferred to polyvinylidene difluoride (PVDF) membranes (Millipore, Billerica, MA, United States). The membranes were incubated with primary antibody against EV71 VP2 (Genetex), followed by incubation with secondary antibody conjugated to an infrared dye (Li-Cor Biosciences, Lincoln, NE, United States). The membranes were visualized using an Odyssey infrared imaging system (Li-Cor Biosciences) as previously described (Wang et al., 2019).

### Evolutionary Relationships of Enteroviral Amino Acid Sequences

In total, 29 selected human enterovirus whole protein amino acid sequences were retrieved from Uniport. The evolutionary history was inferred using the neighbor-joining method (Saitou and Nei, 1987). The bootstrap was set at 1000. The evolutionary distances were computed using the Poisson correction method and are presented in units of the number of amino acid substitutions per site. Evolutionary analyses were conducted with MEGA X software (Kumar et al., 2018).

### Statistical Analysis

Statistical analysis was performed using a two-tailed unpaired t-test in GraphPad Prism software (La Jolla, CA, United States). The data are presented as the means ± standard deviations (SDs; *n* = 3 or as otherwise indicated). All experiments were repeated at least twice.

## Results

### Remdesivir (GS-5734) Is a Potent Antiviral Against EV71

**FIGURE 1:**
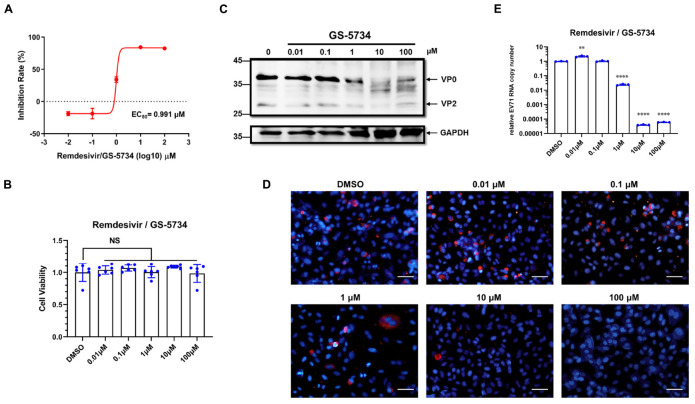
Remdesivir potently inhibits EV71 infection without significant cytotoxicity. **(A)** HeLa cells were infected with EV71 (MOI = 1) and treated with varying concentrations of remdesivir at 1 hpi. The viral RNA copy number was quantified by qRT-PCR at 24 hpi (mean ± SD, *n* = 3). **(B)** Cell viability after culture with different doses of remdesivir at 24 hpi assessed using a CCK8 assay (mean ± SD, *n* = 6). **(C)** Western blot of EV71 VP2 protein expression in HeLa cells. HeLa cells were infected with EV71 (MOI = 0.5). After adsorption, the cells were treated with the indicated concentrations of remdesivir and collected at 24 hpi. Then, the proteins were resolved by SDS-PAGE and western blotting. **(D)** Fluorescence microscopy examination of EV71-infected HeLa cells treated with different amounts of remdesivir (24 hpi). The dsRNA-specific antibody J2 (red) was used for staining viral dsRNA replication intermediates, and nuclei were labeled with Hoechst 33258 (blue). Bar = 100 μm. **(E)** Cell culture supernatants collected from **(A)** were used to infect HeLa cells, and after 1 h of adsorption at 37°C, fresh medium was added. At 24 hpi, cells were collected and subjected to viral RNA copy number quantification by qRT-PCR (mean ± SD, *n* = 3). NS, *P* > 0.05, *P < 0.05, **P < 0.01, ***P < 0.001, and ****P < 0.0001.

Previous studies have reported that remdesivir (GS-5734) inhibits different viruses with different efficacies. To determine whether remdesivir inhibits EV71 replication, we first sought to investigate the antiviral activity of remdesivir against EV71 in a broad dilution range. Based on data from other viruses, we chose five concentrations, ranging from 0.01 μM to 100 μM. We first infected HeLa cells with EV71 (MOI = 1) for 1 h. Then, the inoculum was removed, and medium with remdesivir at varying concentrations was added. After 24 h of treatment, cellular RNA was isolated and subjected to qRT-PCR analysis. Remdesivir showed a dose-dependent inhibition of EV71 replication (Figure 1A, EC_50_ = 0.991 μM) and had little effect on cell viability (Figure 1B), although EV71 replication seemed slightly enhanced at low remdesivir concentrations (0.01 ∼ 0.1 μM). Moreover, viral protein synthesis was inhibited by remdesivir treatment (Figure 1C). During positive-sense RNA virus replication, dsRNA is present in the cellular replication site of the virus and can be detected by dsRNA-specific antibodies. A noticeable reduction in dsRNA staining was discovered in the remdesivir-treated groups (Figure 1D). In addition, the number of secreted virions in the culture supernatant was reduced upon remdesivir treatment (Figure 1E), with an EC_50_ = 0.203 μM. Taken together, these results suggest that remdesivir potently inhibits EV71 replication without affecting cell viability.

### Remdesivir Inhibits EV71 Replication Post Virus Entry

Since remdesivir inhibits the replication of other viruses through targeting of viral RdRp, we sought to rule out the effects of remdesivir on virus entry. To this end, we performed experiments using different methods, as follows.

**FIGURE 2:**
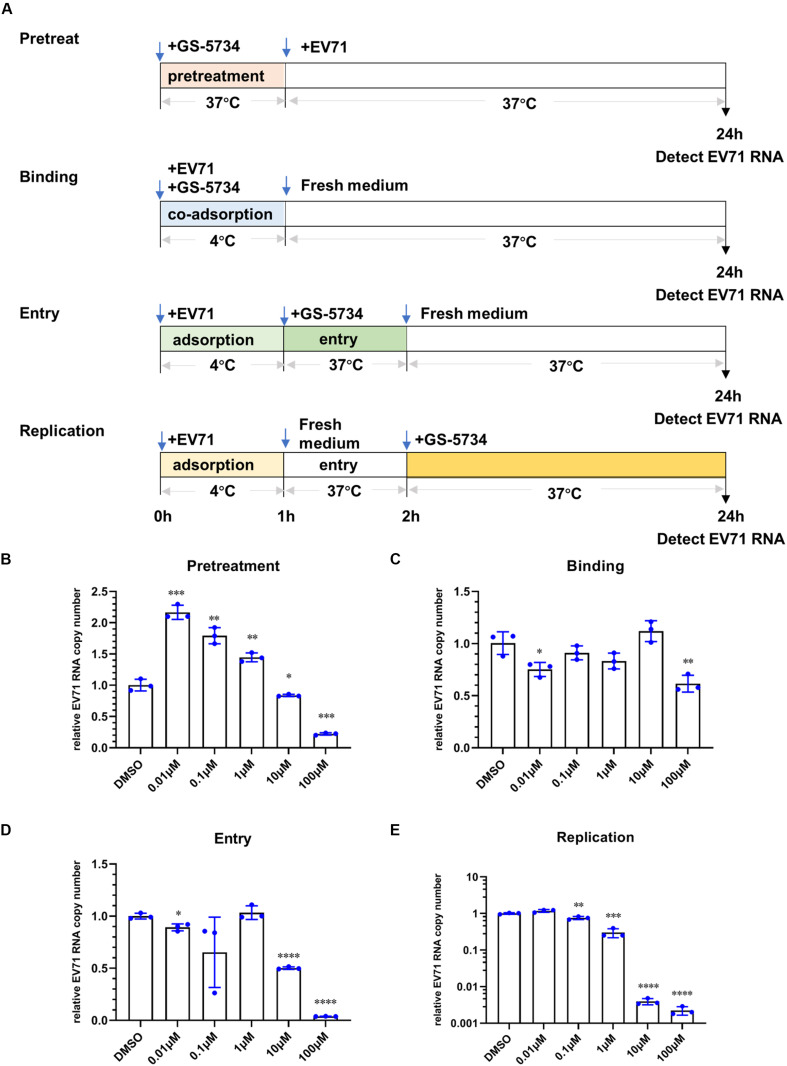
Remdesivir inhibits EV71 replication post virus entry. **(A)** Schematic representation of the different treatment methods. Cells were pretreated with remdesivir **(B)**, coadsorbed remdesivir and EV71 **(C)**, remdesivir after EV71 adsorption that was removed after virus entry **(D)**, or remdesivir after virus entry **(E)**, and at 24 hpi, the EV71 viral RNA copy number was quantified by qRT-PCR (mean ± SD, *n* = 3). *P < 0.05, **P < 0.01, ***P < 0.001, and ****P < 0.0001.

First, cells were pretreated with remdesivir for 2 h, and then, EV71 was adsorbed for 1 h at 4°C (Figure 2A). Intriguingly, after pretreatment, remdesivir slightly enhanced EV71 replication at low concentrations and inhibited virus replication at 100 μM (Figure 2B); similar results were observed for remdesivir-treated SARS-CoV-2 (Wang et al., 2020). Although we cannot find an explanation for the recent phenomenon, the latter inhibition may be because the removal of remdesivir was not sufficient or the intracellular level of remdesivir was still enough to inhibit EV71 replication.

To study whether remdesivir affects EV71 binding to viral receptors on the cell surface, we mixed EV71 and remdesivir and coadsorbed them to HeLa cells for 1 h at 4°C. Then, the mixture was discarded, and the cells were washed with DPBS to remove any unabsorbed EV71. Next, fresh medium was added, and the culture plate was transferred to a 37°C incubator until 24 h hpi (Figure 2A). As depicted in Figure 2C, remdesivir did not inhibit EV71 binding to cells.

To investigate whether remdesivir affects EV71 entry into the cell, EV71 was first added to HeLa cells for 1 h at 4°C. Then, the unbound virions were discarded, and the cells were washed with DPBS. Next, medium containing remdesivir at different concentrations was added, the plate was transferred to a 37°C incubator to initiate virus entry for 1 h (Figure 2A), and then, the medium was refreshed. As expected, remdesivir did not affect EV71 entry into the cell at low concentrations (Figure 2D). The reduction in EV71 RNA at high remdesivir levels may be due to insufficient removal of the drug.

As a control, the effects of remdesivir on viral replication were determined by adding fresh medium containing remdesivir after EV71 binding (4°C) and entry (37°C), and culturing cells until 24 hpi (Figure 2A). As expected, under this condition, remdesivir severely inhibited EV71 replication, and even abolished EV71 transcription at high concentrations (Figure 2E).

Combined, these data illustrate that remdesivir effectively inhibits the proliferation of EV71 post virus entry.

### Remdesivir Inhibits EV71 Replication at the Viral Transcription Stage and Exhibits Broad-Spectrum Inhibition of Enterovirus Replication

**FIGURE 3:**
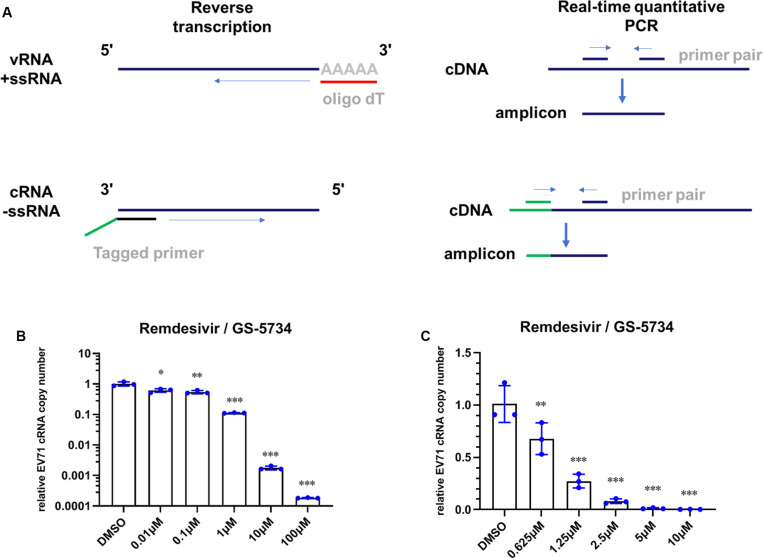
Remdesivir inhibits EV71 replication at the viral transcription stage. **(A)** Schematic representation of reverse transcription and PCR protocol for conventional real-time PCR (top) and tagged primer-mediated qRT-PCR (bottom). The tag within the tagged RT primer is shown in green, and the amplified products contained a portion of the tag and cDNA. **(B,C)** Synthesis of EV71 cRNA (normalized to the actin expression level) was inhibited by remdesivir (mean ± SD, *n* = 3). *P* > 0.05, *P < 0.05, **P < 0.01, and ***P < 0.001.

To investigate the possible mechanism by which remdesivir inhibits EV71 infection, we first sought to detect the level of EV71 cRNA upon remdesivir treatment. To this end, we designed a specific primer containing a unique tag for reverse transcription, and a forward qRT-PCR primer was designed to specifically target the tag (Figure 3A) (Kawakami et al., 2011). The different levels of EV71 cRNA were determined by qRT-PCR, and after normalization of the EV71 level cRNA to actin expression, a reduction in EV71 cRNA was observed after remdesivir treatment (Figures 3B,C). Therefore, remdesivir reduced both cRNA and vRNA synthesis of EV71. Enterovirus cRNA and vRNA are both synthesized by 3D/RdRp. Within the palm subdomain, three motifs (A, B, and C) are highly conserved. In particular, motif B forms a “loop-α-helix” structure to assist binding of template RNA, and an Asn residue is involved in substrate discrimination, which preferentially selects ribonucleoside triphosphates over dNTPs (Shu and Gong, 2016; Warren et al., 2016; Peersen, 2017). A previous study superimposed different RNA virus RdRp sequences, revealing that rhinoviruses, flaviviruses, and noroviruses share high similarity between nucleotide binding domains (Lo et al., 2017). Hence, the antiviral spectrum of remdesivir may be extended to other enteroviruses.

**FIGURE 4:**
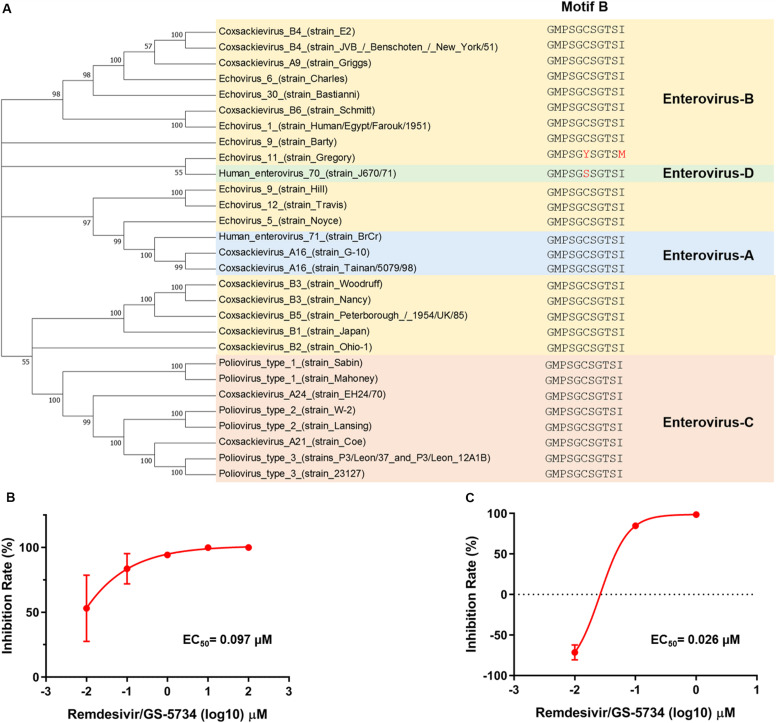
Remdesivir exhibits broad-spectrum inhibition of enterovirus replication. **(A)** Phylogenetic analyses of the human enterovirus amino acid sequence. EV-A is marked in light blue, EV-B is marked in gold, EV-C is marked in orange, and EV-C is marked in green. CVB3 **(B)** and EV70 **(C)** RNA synthesis was inhibited by remdesivir (mean ± SD, *n* = 3). *P < 0.05, ***P < 0.001, and ****P < 0.0001.

Based on protein amino acid sequences retrieved from Uniport, motif B is highly conserved among the four human enterovirus species (Figure 4A). To further verify whether remdesivir also inhibits the replication of other enteroviruses, we selected two enteroviruses stocked in the laboratory and found that remdesivir also showed potent efficacy toward CVB3 (Figure 4B) and EV70 (Figure 4C), with EC_50_ = 0.097 μM, and EC_50_ = 0.026 μM, respectively. These data demonstrated that remdesivir exhibits broad-spectrum antiviral efficacy against enterovirus.

## Discussion

Human enteroviruses can cause a wide range of diseases in humans, from mild respiratory illness to myocarditis and from HFMD to severe aseptic meningitis. The most harmful virus, PV, is possibly the next virus that will be eradicated after variola major. The top two HFMD causative pathogens are EV71 and CAV16 (Lee, 2016). EV71 is responsible for severe neurological complications and is considered the most critical enterovirus in the post-polio era. However, the lack of specific antivirals makes enterovirus infection treatment largely dependent on supportive therapy; thus, pan-enterovirus antivirals are needed.

Here, we discovered that a phosphoramidate prodrug of Nuc, remdesivir, which has already been shown to be effective against EBOV (Jacobs et al., 2016; Warren et al., 2016; Tchesnokov et al., 2019), CoV (Sheahan et al., 2017; Agostini et al., 2018), and NiV (Lo et al., 2017; Lo et al., 2019), exhibits prominent efficacy against EV71. Further experiments revealed that the antiviral effects of remdesivir begin after virus entry and include inhibition of viral cRNA and vRNA synthesis.

The possible mechanism by which remdesivir inhibits RNA virus replication has been elucidated in *Paramyxoviridae* and *Filovirus*. For RSV and NiV, remdesivir causes premature termination of nascent RNA transcripts (Warren et al., 2016; Jordan et al., 2018); a similar result was observed for EBOV RdRp. In addition, remdesivir caused a delayed chain termination that could not be overcome by the addition of higher concentrations of nucleotides (Tchesnokov et al., 2019). Although the precise mechanism by which remdesivir inhibits enterovirus replication still needs further investigation, using cRNA PCR, we noticed that EV71 cRNA was nearly undetectable after exposure to high concentrations of remdesivir. Hence, 3D may incorporate remdesivir into its nascent RNA and cause subsequent inhibition of EV71 cRNA synthesis.

Besides, RdRp motif B within the 3D protein of different enteroviruses is highly conserved (Bruenn, 2003; Shu and Gong, 2016; Peersen, 2017), and remdesivir inhibited CVB3 and EV70 replication, which represent human enteroviruses B and D, respectively. Consequently, remdesivir can be further developed as a pan-enterovirus antiviral.

One limitation of our study is the lack of an *in vivo* efficacy evaluation of remdesivir due to the lack of a suitable animal model. For EV71, two conventional models are used: a mouse-adapted virus strain that can cause neurological symptoms after infection (Wang et al., 2011; Chang et al., 2015) and intracranial infection of newborn suckling mice (Lee et al., 2014). However, our laboratory does not possess the mouse-adapted strain, and the newborn mouse model is ineffective for evaluating the efficacy of antivirals. In the most prevalent suckling mouse model, it is challenging to compare antiviral efficacy due to an excessive number of variations. Future studies can be adopted by utilizing Scavenger Receptor Class B Member 2 (SCARB2), a receptor of EV71, in transgenic mice to evaluate the effect of remdesivir on EV71 (Chang et al., 2013; Fujii et al., 2013). Regarding the other receptor, P-selectin glycoprotein ligand-1 (PSGL-1), transgenic mice are insufficient for evaluation of EV71 infection (Liu et al., 2012). The *in vivo* efficacy of remdesivir against other enteroviruses might be evaluated in existing enteroviral mouse models, e.g., CVB3.

An intriguing result observed in this study was that remdesivir slightly enhanced EV71 replication at low concentrations (0.01 ∼ 0.1 μM). Moreover, this effect is likely related to remdesivir pretreatment, which may affect subsequent viral entry steps. Additionally, remdesivir was found to enhance SARS-CoV-2 entry compared to chloroquine slightly. The mechanism by which remdesivir enhances virus replication after pretreatment needs further investigation. However, this study suggests the possibility that combining remdesivir with other virus entry inhibitors, e.g., neutralizing antibody or nafamostat, may be efficient for the treatment of viral diseases by compensating for the virus replication enhancing effects of remdesivir. Also, these findings should be taken into account when considering the remdesivir research during the current SARS-CoV-2 pandemic.

In summary, remdesivir is a potent inhibitor of replication of EV71 and other enteroviruses in cells. Hence, future studies may combine accurate diagnosis and effective antivirals to prevent possible neurological disorders resulting from enterovirus infection, especially in EV71-induced cases.

## Data Availability Statement

The datasets generated for this study can be found in NCBI under accession number MK904809. All other datasets generated for this study are included in the article/supplementary material.

## Author Contributions

WY, YL, and FZ conceived of the study. WY wrote the manuscript. WY performed most of the experiments. MY, YD, and CY provided experimental technique support. DW executed the pharmaceutical data analysis. YW, HL, HM, HZ, LZ, LQ, YY, LC, and XL provided experimental materials. ZX, YL, and FZ provided administrative support. WY, ZX, YL, and FZ provided financial support. WY, YL, and FZ checked and finalized the manuscript.

## Conflict of Interest

The authors declare that the research was conducted in the absence of any commercial or financial relationships that could be construed as a potential conflict of interest.
