# Corrigendum: Remdesivir (GS-5734) Impedes Enterovirus Replication Through Viral RNA Synthesis Inhibition

**DOI:** 10.3389/fmicb.2020.621197

**Published:** 2020-11-23

**Authors:** Wei Ye, Min Yao, Yangchao Dong, Chuantao Ye, Dan Wang, He Liu, Hongwei Ma, Hui Zhang, Libin Qi, Yuewu Yang, Yuan Wang, Liang Zhang, Linfeng Cheng, Xin Lv, Zhikai Xu, Yingfeng Lei, Fanglin Zhang

**Affiliations:** ^1^Department of Microbiology, School of Preclinical Medicine, Fourth Military Medical University, Xi'an, China; ^2^Department of Infectious Diseases, Tangdu Hospital, Fourth Military Medical University, Xi'an, China; ^3^Second Affiliated Hospital, Xi'an Medical University, Xi'an, China; ^4^Cadet Brigade, School of Preclinical Medicine, Fourth Military Medical University, Xi'an, China

**Keywords:** Remdesivir (GS-5734), antivirals, EV71, vRNA, cRNA, enterovirus

In the original article, there was a mistake in Figure 1E as published. The corrected Figure 1E appears below.

**Figure 1:**
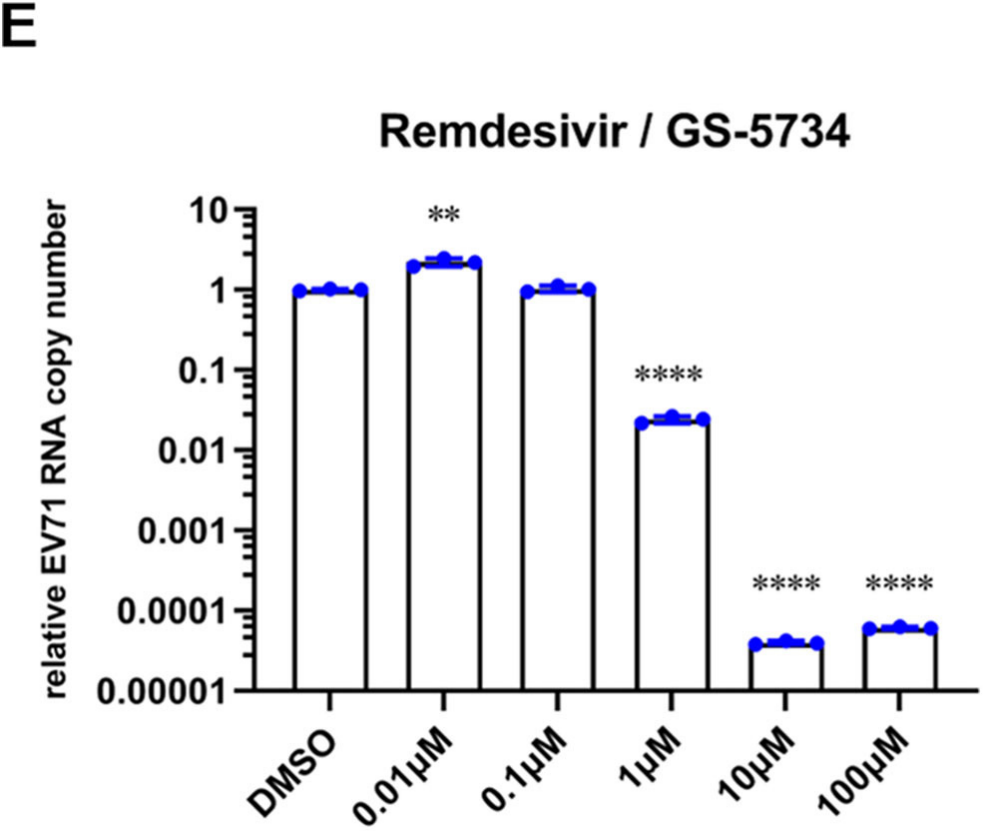


The authors apologize for this error and state that this does not change the scientific conclusions of the article in any way. The original article has been updated.

